# CHD5, a Brain-Specific Paralog of Mi2 Chromatin Remodeling Enzymes, Regulates Expression of Neuronal Genes

**DOI:** 10.1371/journal.pone.0024515

**Published:** 2011-09-13

**Authors:** Rebecca Casaday Potts, Peisu Zhang, Andrea L. Wurster, Patricia Precht, Mohamed R. Mughal, William H. Wood, Yonqing Zhang, Kevin G. Becker, Mark P. Mattson, Michael J. Pazin

**Affiliations:** 1 Laboratory of Molecular Biology and Immunology, National Institute on Aging Intramural Research Program, National Institutes of Health, Baltimore, Maryland, United States of America; 2 Laboratory of Neuroscience, National Institute on Aging Intramural Research Program, National Institutes of Health, Baltimore, Maryland, United States of America; 3 Research Resources Branch, National Institute on Aging Intramural Research Program, National Institutes of Health, Baltimore, Maryland, United States of America; Université Paris-Diderot, France

## Abstract

CHD5 is frequently deleted in neuroblastoma and is a tumor suppressor gene. However, little is known about the role of CHD5 other than it is homologous to chromatin remodeling ATPases. We found CHD5 mRNA was restricted to the brain; by contrast, most remodeling ATPases were broadly expressed. CHD5 protein isolated from mouse brain was associated with HDAC2, p66ß, MTA3 and RbAp46 in a megadalton complex. CHD5 protein was detected in several rat brain regions and appeared to be enriched in neurons. CHD5 protein was predominantly nuclear in primary rat neurons and brain sections. Microarray analysis revealed genes that were upregulated and downregulated when CHD5 was depleted from primary neurons. CHD5 depletion altered expression of neuronal genes, transcription factors, and brain-specific subunits of the SWI/SNF remodeling enzyme. Expression of gene sets linked to aging and Alzheimer's disease were strongly altered by CHD5 depletion from primary neurons. Chromatin immunoprecipitation revealed CHD5 bound to these genes, suggesting the regulation was direct. Together, these results indicate that CHD5 protein is found in a NuRD-like multi-protein complex. CHD5 expression is restricted to the brain, unlike the closely related family members CHD3 and CHD4. CHD5 regulates expression of neuronal genes, cell cycle genes and remodeling genes. CHD5 is linked to regulation of genes implicated in aging and Alzheimer's disease.

## Introduction

A role for CHD5 in cancer was first suggested by genetic mapping studies in neuroblastomas [1], [2]. Neuroblastomas frequently harbor a deletion of the short arm of human chromosome 1, and the region shared by most deletions includes the CHD5 gene [1], [3]. CHD5 was confirmed as the tumor suppressor in this region, as depletion of CHD5 phenocopied the proliferative defects found with deletions engineered in mice [2], [4]. Depletion of CHD5 reduced expression of another tumor suppressor, p19Arf, suggesting that CHD5 mediates its tumor suppressive activity through modulation of the p19arf/p53 pathway [4]. Subsequently, CHD5 has been reported to be mutated, deleted or silenced in a variety of human cancers including glioma, leukemia/lymphoma, melanoma, breast, prostate, ovarian and gastric cancers [5], [6], [7], [8], [9], [10], [11], [12], [13]. CHD5 expression has been suggested to serve as a biomarker for positive outcomes in neuroblastoma patients [14].

Chromodomain/helicase/DNA-binding domain (CHD) proteins are found in eukaryotes from yeast through humans [15], [16]. All CHD members contain two N-terminal chromodomains, a helicase-like ATPase motif associated with nucleosome remodeling, and a less well-defined C-terminal DNA binding domain. The tandem chromodomains of CHD1 specifically recognize H3K4Me3 and can facilitate the recruitment of post-transcriptional initiation and splicing factors [17], [18]. The human CHD family is often divided by sequence homology: subfamily I (CHD1 and CHD2), subfamily II (CHD3 and CHD4) and subfamily III (CHD6, CHD7, CHD8, CHD9); CHD5 has been grouped with CHD 6–9 by some authors, and CHD 3–4 by others [15], [16], [19], [20]. Many remodeling ATPases are expressed ubiquitously in the body plan. CHD5 is unusual in that its expression is reported to be limited to the developing brain, adult brain and the adrenal gland, suggesting a potential role in the development or function of the neural system [21]. Deletion of a region of chromosome 1 near CHD5 has been linked to intellectual impairment [22]. However, the role of CHD5 in brain development and function remains to be determined.

CHD3 and CHD4, also known as Mi-2alpha and Mi-2ß, are found in multiprotein chromatin remodeling complexes named NuRD [23], [24], [25], [26], [27], [28]. In addition to the ATPase activity of the CHD subunit, NuRD complexes include the histone deacetylases HDAC1 and HDAC2. NuRD complexes include a methyl CpG binding protein (MBD2 or MBD3), adapter proteins (RbAp46 and/or RbAp48), p66, and a metastasis associated protein (MTA1, 2 or 3).

CHD proteins have been demonstrated to regulate gene expression, with CHD3 and CHD4 being the best-studied examples [24]. NuRD is often described as a transcriptional repressor, in part because of the presence of histone deacetylase activity. However, NuRD complex activity results in divergent outcomes for two target genes in developing lymphocytes, mb-1 and CD4: NuRD inhibits mb-1 transcription and activates CD4 transcription [29], [30]. NuRD is also an activator and repressor during blood development [31]. This behavior is reminiscent of the mammalian ISWI remodelers, which are also often thought of as repressors, yet appear to possess activation potential as well [32], [33], [34]. How the NuRD complex can differentially regulate gene transcription remains an open question but it has been suggested to be an outcome of a regulated shift in the composition of NuRD components or through the association with other transcriptional regulators. Genome-wide analysis of Mi2 binding in *D. melanogaster* revealed association with regions that appeared to be enhancers and promoters [35]. Less is known about the potential chromatin remodeling and gene regulatory functions of CHD5–CHD9. Whether CHD5 exists in a multi-protein complex and functions to regulate gene expression in the brain has not been reported.

Remodeling of chromatin structure is an important determinant of cell fate decisions and function in the nervous system. In particular, ATP-dependent remodeling has been shown to be critical for the development of invertebrate and vertebrate nervous systems [19], [36], [37]. The best-characterized example is SWI/SNF, containing the BRG1 and BRM ATPases. The SWI/SNF ATPases are ubiquitous, though the accessory proteins in the complex are developmentally regulated. During the transition from neural progenitor to post-mitotic neuron, BAF45a and BAF53a are replaced by BAF45b and BAF53b to assemble the brain-specific nBAF form of the SWI/SNF complex [38]. nBAF is critical in neuron-specific function as BAF53b-deficient mice have a defect in neuron dendrite outgrowth [39]. In addition to ATP-dependent remodeling, HDAC-inhibitor studies revealed a role for chromatin remodeling involving histone acetylation in synaptic plasticity and learning behaviors [40], [41]. Studies on HDAC2-deficient mice implicated this particular deacetylase as a direct negative regulator of learning and memory [42].

Here, we examine CHD5 in rodent brain and neurons. We found CHD5 was present in a multiprotein NuRD-like complex. CHD5 was expressed in several brain regions, and CHD5 was found in neurons. Depletion of CHD5 from primary neurons revealed genes that were activated and repressed by CHD5. The targets included genes that were previously identified as important for aging, Alzheimer's disease, and neuronal development. Binding of CHD5 to some of these target genes in intact cells suggested they were directly regulated by CHD5.

## Materials and Methods

### Protein extracts from rat brain

Rat brain was dissected to separate the cortex, cerebellum, hippocampus, brain stem and striatum. Nuclear extracts were made from each rat brain section as described for mouse brain nuclear extracts (Methods S1). 15 ug of nuclear extract from each brain region was analyzed by SDS-PAGE, transferred to PVDF membrane, and detected by immunoblotting using anti-HDAC-2 (Abcam ab7029), and reprobed with anti-CHD5 (HD5A-E Day 77). Animal approval for rats was from the NIA ACUC, protocol ASP-289-MPM, and all experiments conform to the relevant regulatory standards.

### Staining of brain sections

CHD5 staining of the rat hippocampus was done as described [43] using anti-CHD5 antibody (HD5A-A Day 77) or pre-immune serum at 1∶2000.

### Staining of primary neurons

Rat primary neurons were harvested from E18 rats as described [44]. Cells were plated on PEI coated coverslips and were grown in Neural Basal Medium (+B27) for nine days *in vitro* (DIV). Cells were fixed in 100% cold methanol for 15 minutes at −20°C, pre-incubated with blocking buffer (2% nonfat powdered milk, 2% normal serum in PBS) for 1 hour. Cells were incubated with primary antibodies diluted 1∶1000 in blocking buffer for 72 hours at 4°C. Primary antibodies used were: anti-CHD5 (HD5A-A Day 77) and anti Tuj1, mouse monoclonal anti Beta-Tubulin isotype III (Sigma #T8660). Secondary antibodies were Alexa 568 for Tuj1 and Alexa 488 for CHD5. Cells were counterstained with DAPI in PBS for 10 minutes.

### Identification of proteins associated with CHD5

CHD5 antibody (HD5A-A Day 77) or pre-immune serum was crosslinked to protein A beads as described [45]. Mouse brain nuclear extract (12 mg) was diluted 1∶4 in RIPA Buffer (50 mM Tris, pH 7.5, 150 mM NaCl, 1% NP40, 0.5% sodium deoxycholate, 0.1% SDS), antibody/protein A beads were added and incubated overnight at 4°C. The beads were pelleted, and washed 4 times with TX-100 Buffer [0.02 M Tris/HCl pH 8.0, 0.137 M NaCl, 10% glyceol (v/v) and 1% Triton X-100 (v∶v)] with protease inhibitors. A final wash was performed with TE. The immunoprecipitated proteins were eluted from the beads using 0.1 M Glycine pH 2.5. The immunopurified proteins were analyzed by SDS-PAGE on an 8% polyacrylamide gel. Protein bands were visualized by silver staining (BioRad) or Colloidal Coomasie Blue staining. Protein bands were excised from Coomasie-stained gels and were sequenced (ProtTech Inc). Animal approval for mice was from the NIA ACUC, protocol ASP-365-MJP-Mi, and all experiments conform to the relevant regulatory standards.

### Co-immunoprecipitation of proteins

Mouse brain nuclear extract was immunopurified as described above except that these antibodies were crosslinked to the protein A beads: anti-HDAC1 (Santa Cruz, sc-7872; Upstate, 06-720), anti-HDAC2 (Abcam ab7029-50), anti-p66 Beta (Bethyl, A301-281A). Other CHD5-interacting proteins were immunopurified using the previously described protocol with these modifications: the antibodies were not crosslinked to the protein A beads and the nuclear extract was diluted into CoIP Buffer II (100 mM NaCl, 20 mM Hepes pH 7.5, 1 mM EDTA, 10% glycerol, 0.1% NP40). Immunopurified proteins were analyzed by SDS-PAGE using 8% Polyacrylamide gels. Immunoblot analysis was performed probing for CHD5 using CHD5 antisera (HD5A-E Day 77).

### Gel filtration analysis of CHD5

Mouse brain nuclear extract was dialyzed against PBS (MWCO 12–14,000, 6.4 mm diameter Spectrum Laboratories 132 676), centrifuged twice at 100,000× g for 10 minutes at 4°C, then 0.2 mg was loaded onto a Sephadex 200 Column (Amersham) and 0.5 mL eluted fractions were collected and analyzed by SDS-PAGE and western blot analysis. Sizing standards were run according to the manufacturer's instructions (Amersham High Molecular Weight Gel Filtration Calibration Kit, 17-0441-01).

### CHD5 protein depletion using lentiviral-delivered shRNA

CHD5 KD shRNA sequences (Table S5) were designed according to the instructions for the pLKO.1 system (Addgene). Control shRNA virus was plasmid 1864 from Addgene, as described [46]. Virus was packaged using HEK-293T cells, pLKO.1 vector with shRNA inserts for CHD5, psPAX2 and pMD2.G. 48 hours after transfection of 293 cells, medium containing virus was filtered (0.45 micron), then applied to primary cortical neurons one day after the neurons were plated (DIV 1) for 6 hours. Medium was removed, and replaced with Neural Basal Medium, and the cells were cultured until RNA was harvested at day 5, 9, or 12 (DIV 5, 9 or 12).

### Validation of Microarray

RNA was harvested from primary cortical neurons infected with the indicated lentivirus, as described above, at the indicated times. RNA was isolated using RNAeasy Kit (Qiagen), cDNA was synthesized using Iscript (BioRad). mRNA was quantified using real time PCR and normalized to Actin (Actb), a housekeeping gene; similar results were obtained normalizing to other housekeeping genes such as Tbp [47] or Gapdh. Primer sequences are listed in Table S5.

### ChIP (chromatin immunoprecipitation)

ChIP was performed essentially as described [32], [48], [49], [50], [51], with the following changes. Approximately 3×10^6^ primary cortical neurons were cross-linked with 1% formaldehyde for 10 minutes, then quenched with glycine. Cells were scraped into PBS, collected by centrifugation, and lysed in 400 ul SDS Lysis Buffer (50 mM Tris-HCl pH 8, 1% SDS, 10 mM EDTA). Nuclei were not treated with MNase. Sonication was performed using a bath sonicator (Bioruptor; Diagneode). Mouse brain ChIP was performed as described [32], [52]. CHD5 ChIP was performed using CHD5 antisera on day 9 (HD5A-A Day 77). Rat and mouse primer sequences are listed in Table S5.

## Results and Discussion

### CHD5 mRNA is preferentially found in brain

**Figure 1:**
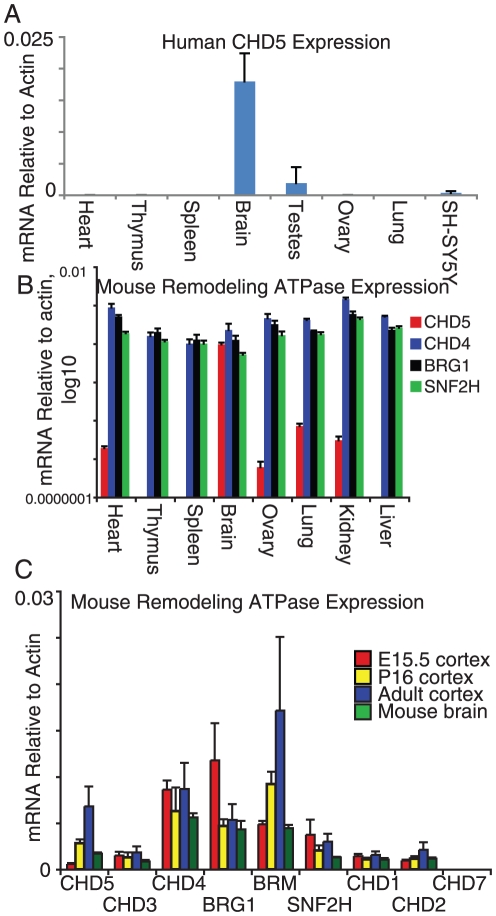
CHD5 expression is restricted to brain. A,B) RNA from human (A) or mouse (B) organs was used to synthesize cDNA, which was then quantified by real-time PCR. Signal was normalized to the expression of the housekeeping gene Actin. The bars indicate the averages and standard deviations of three experiments. A) SH-SY5Y indicates the human neuroblastoma cell line SH-SY5Y; neuroblastoma cells do not express the tumor suppressor CHD5. B) expression for each remodeling enzyme was normalized to the expression level in mouse brain; thus, a value of 1 means the expression for that enzyme in that tissue is the same as brain. CHD5 was not detected in mouse Th1 and Th2 cells (primary naïve CD4+ T lymphocytes differentiated in culture to the Th1 or Th2 fate; data not shown). C) RNA obtained from dissected mouse brain (brain at E15.5, cortex at P16, and adult cortex) as well as commercial mouse brain RNA was used to synthesize cDNA, which was then quantified by real-time PCR for each of the indicated remodeling enzyme ATPases. Signal was normalized to the expression of the housekeeping gene Actin. Mouse brain at E15.5 is essentially cortical neurons. The bars indicate the averages and standard deviations of three experiments.

CHD5 expression was initially reported to be limited to brain, though others have found CHD5 expression elsewhere, so there is some controversy about sites of CHD5 expression [4], [7],[9],[13],[21]. We examined CHD5 mRNA in mouse and human organs, and found that CHD5 mRNA was abundant in brain, approximately 7 fold lower in mouse testes, and 2–4 orders of magnitude lower in all other tested mouse and human organs (Figure 1a,b). Little if any CHD5 mRNA was found in the neuroblastoma cell line SH-SY5Y; this is a negative control, as neuroblastoma cells generally do not express the neuroblastoma tumor suppressor gene CHD5. By contrast, for the same mouse mRNA samples, the ratio of the highest to lowest expression for CHD4, BRG1 and SNF2H (three other remodeling ATPases) was about 5 fold, indicating they were broadly expressed (Figure 1b). We place CHD5 along with CHD4 in the Mi2 group of remodeling ATPases by sequence homology (see below), thus the contrast in expression was specific to CHD5, not a property of the Mi2 family. CHD5 mRNA was detected at E15.5, when the brain is mostly neurons with relatively few glial cells, and increased after birth and to adulthood (Fig. 1c). RNA encoding other remodeling enzymes was also detected, including CHD3 and CHD4, sequence homologs of CHD5 (Fig. 1c). Again, the expression of CHD5 was very different than the expression of the paralogs CHD3 and CHD4.

### CHD5 is a nuclear protein found in neurons

**Figure 2:**
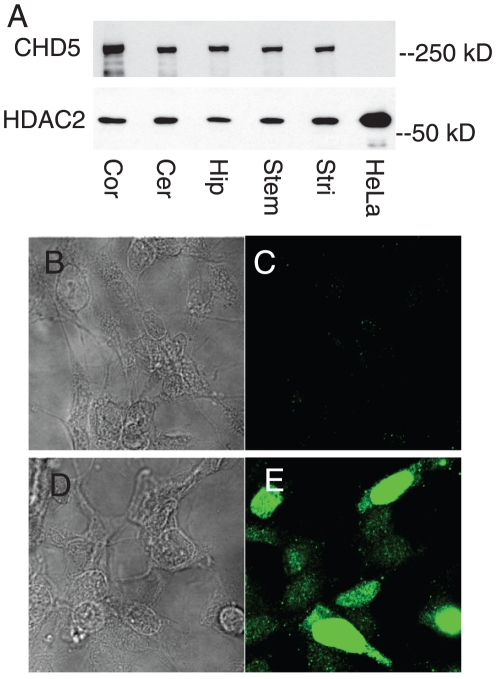
CHD5 protein is expressed in several brain regions. A) The indicated regions were dissected from rat brain. Nuclear extracts were made from rat brain and HeLa cells. Equal amounts of protein were loaded in each lane. CHD5 was detected by immunoblotting with HD5A-E day 77. The same filter was reprobed for HDAC2 as a loading control. B–E) CHD5 antibody specificity. SH-SY5Y neuroblastoma cells were transfected with human CHD5 cDNA (D, E) or control (B, C) using lipofectamine2000. Cells were fixed with methanol −20°C 15 minutes, visualized by phase contrast (B, D) or were stained for CHD5 protein (primary is 1∶1000 CHD5 antisera HD5A-A day 77, secondary is Alexa 488 anti-rabbit).

**Figure 3:**
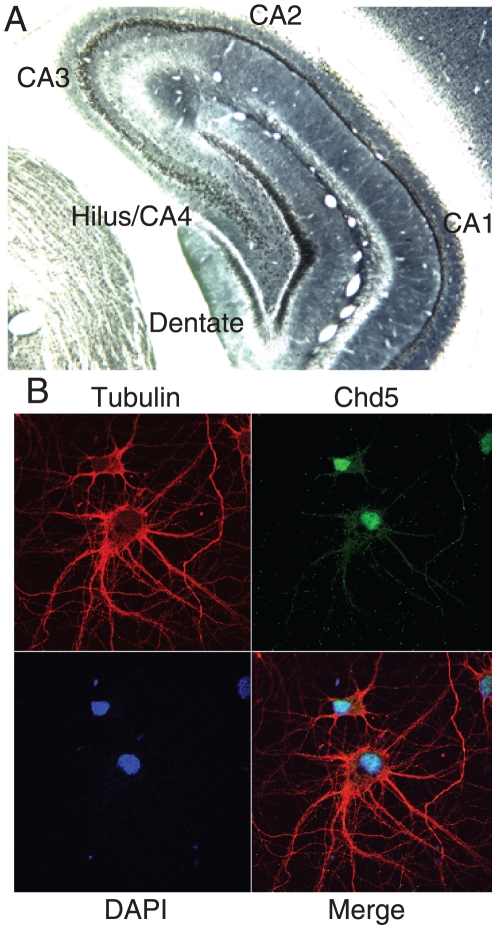
CHD5 protein is a nuclear protein in rodent brain and cultured neurons. A) Rat brain sections were prepared, stained with CHD5 antisera HD5A-A day 77, and protein was visualized using immunohistochemistry. B) Neurons harvested from E18 rats were grown on coverslips, and stained for CHD5 (Green; Antisera HD5A-A day 77) and a neuronal marker (Red- Tuj1), and counterstained for DNA (Blue-DAPI). Individual and merged panels are shown.

Remodeling ATPases are grouped by homology into subfamilies. Based upon sequence identity and domain structure, CHD5 is a member of the Mi2 group, along with the paralogs CHD3 and CHD4 (Figure S1). CHD5 is most homologous to CHD4, with 70% amino acid identity and identical domain structure (Paired PHD domains, paired chromodomains, SNF-2-like ATPase domain). By comparison, CHD5 shares only 18% identity with SNF2H, an ISWI ATPase, and 18% identity with BRG1, a SWI2/SNF2 ATPase. We made antisera targeted to CHD5 N-terminal fragments (Figure S1b) that specifically recognize CHD5, and used these antisera to test for CHD5 in brain. We found CHD5 protein was expressed in various regions of rodent brain (Figure 2a). By contrast, CHD5 protein was not detected in HeLa (epitheliod), SH-SY5Y (neuroblastoma), NT2 (embryonal carcinoma), and EL4 (T cell) cell lines, consistent with the lack of CHD5 mRNA in these cells (Figure 2a and data not shown). CHD5 staining was not detected in SH-SY5Y cells, a human neuroblastoma line; following transfection with a human CHD5 cDNA, nuclear staining was readily apparent in these cells (Figure 2). This suggested that our antisera were specific for CHD5; we used SH-SY5Y as a negative control because they a cell line with a neuronal phenotype that lack the neuroblastoma suppressor CHD5. CHD5 protein expression was examined in rodent brain sections. CHD5 protein was readily detected at regions rich in neuronal cell bodies, and was not detected in glial cell rich regions, suggesting CHD5 was a neuronal protein found in cell nuclei (Figure 3a), consistent with a recent report [14]. These signals were not detected when CHD5 primary antibody was omitted from the staining procedure (data not shown). Staining of primary rat neurons confirmed CHD5 was predominantly a nuclear protein in neurons (Figure 3b).

Of course, it is not possible to prove a negative proposition. We were not able to detect CHD5 mRNA or protein outside of brain and perhaps testes at levels higher than in our neuroblastoma negative control. It is nonetheless possible that CHD5 below our detection limit has some important functional role in these cells. In addition, we did not test all possible cell types at developmental stages, so there may be other cell types that express CHD5. Examination of published reports of CHD5 expression outside brain, revealed that several measured mRNA and did not detect the protein [4], [7], [9], another reports CHD5 is a cytoplasmic protein [13] and another reports a CHD5-specific antibody detects CHD5 in HeLa cells [53].

### CHD5 is found in a NuRD-like complex

**Figure 4:**
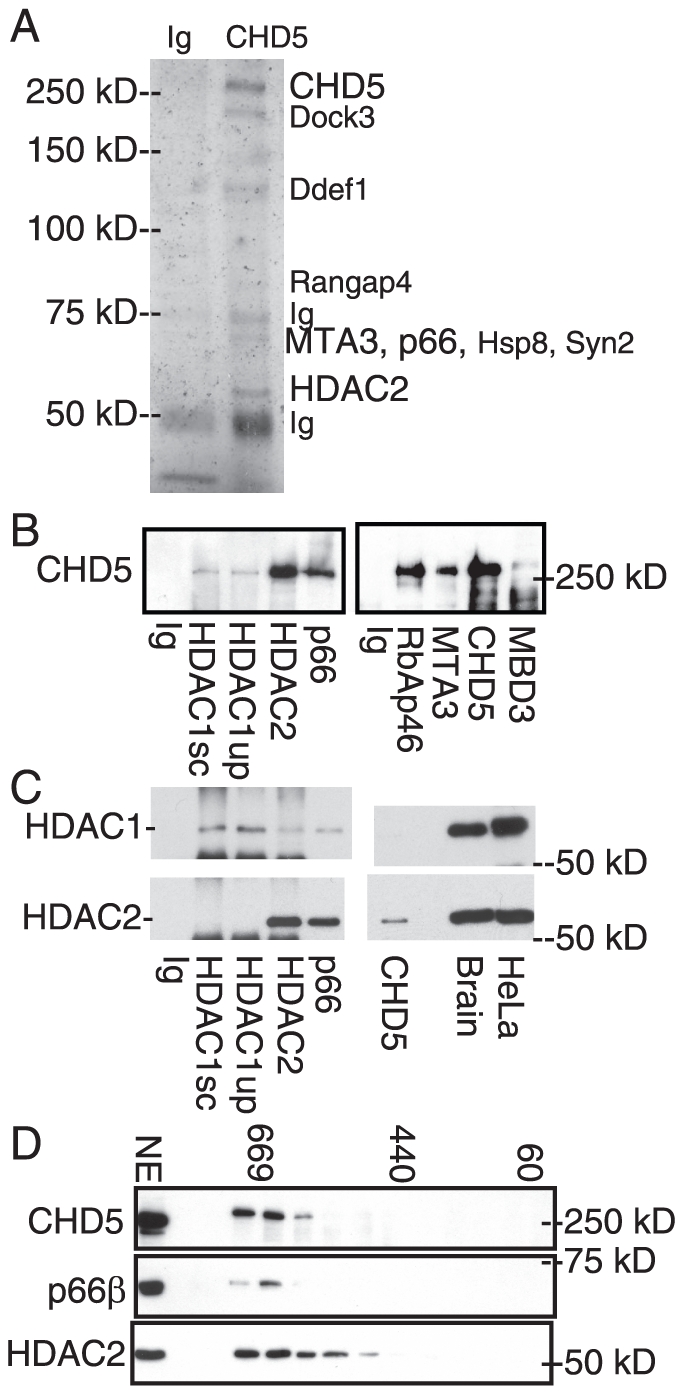
CHD5 protein is found in a NuRD-like complex in mouse brain. A) Proteins were purified from mouse brain nuclear extract using CHD5 antisera HD5A-A day 77 or control IgG crosslinked to Protein A beads. Proteins were analyzed by SDS-PAGE and detected by silver stain. B) Mouse brain nuclear extracts were immunoprecipitated using the antisera indicated below, analyzed by SDS-PAGE, transferred, and detected by immunoblotting using CHD5 antisera. Note different CHD5 antisera were used for IP (HD5A-A day 77) and for immunoblotting (HD5A-E day 77). C) Mouse brain nuclear extracts were immunoprecipitated using the antisera indicated below, analyzed by SDS-PAGE, transferred, and detected by immunoblotting using HDAC1 or HDAC2 antisera. Brain indicates mouse brain nuclear extract; HeLa indicates HeLa nuclear extract. D) Mouse brain nuclear extracts were size-fractionated using gel filtration. Size of fractions was estimated by calibrating the column with standards. Fractions were analyzed by SDS-PAGE, transferred, and detected by immunoblotting with the indicated antisera (for CHD5, antisera HD5A-E day 77 was used). The input nuclear extract (NE) is shown as a positive control.

We asked whether CHD5 was associated with other proteins. Human CHD3 and CHD4 form NuRD-like complexes [24], while Drosophila Mi2 proteins have also been found as free proteins and in complexes without NuRD components [reviewed in 54]. We considered that CHD5 could be in NuRD-like complexes, or perhaps in non-NuRD complexes like the fly homolog. We immunopurified CHD5 from mouse brain nuclear extracts, isolated gel slices, and identified proteins using mass spectrometry. Little, if any, CHD5 was found in the cytoplasmic fraction, consistent with our staining results. Our immunopurification appeared to be specific for CHD5, as sequencing of the band at the expected size for CHD5 revealed the presence of peptides unique to CHD5, while we did not detect peptide sequences from either CHD3 or CHD4 that were absent from CHD5 (Table S1a). Note that CHD3 and CHD4 are identical in size to CHD5, thus cannot be distinguished by electrophoretic mobility. We identified MTA3, p66ß and HDAC1/2 as proteins associated with CHD5 (Figure 4a and Table S1). These components or homologs have been found in NuRD complexes along with the ATPases CHD3 and CHD4 [25], [26], [27], [28], suggesting CHD5 was in a NuRD-like complex.

We used co-immunoprecipitation experiments to further define CHD5-interacting proteins. We confirmed the interaction of MTA3, p66ß, and HDAC2 with CHD5 (Figure 4b). We also detected a strong interaction with RbAp46 (Figure 4b). A weak interaction was found with MBD3; it is not clear whether the lower signal reflects MBD3 abundance, stability of interaction with MBD3 or antibody efficiency. We noted HDAC1 was detected at lower levels than HDAC2 (Figure 4b and Table S1b), suggesting that CHD5 was preferentially interacting with HDAC2. Immunoblotting with HDAC1 instead of CHD5 confirmed HDAC1 was immunoprecipitated with HDAC1 antisera, but not by CHD5 antisera (Figure 4c). Immunoblotting with HDAC2 confirmed that CHD5, p66ß and HDAC2 antisera immunoprecipitated HDAC2. Both HDAC1 and HDAC2 were readily detected in nuclear extracts from mouse brain and HeLa cells (Figure 4c).

We hypothesized that CHD5 might bind HDAC2 with higher affinity, or alternatively cells expressing CHD5 might contain more HDAC2 than HDAC1. We transfected CHD5 cDNA into HeLa cells, which contain HDAC1, HDAC2, CHD3, and CHD4 [26], [27], but lack CHD5 (Figure 2a). CHD5 could interact with both HDAC1 and HDAC2 in these cells, as found for CHD4 (Figure S2). This also indicates the HDAC1 antibody is capable of identifying HDAC1/CHD5 complexes, when present. Others have found that neurons preferentially contain HDAC2 instead of HDAC1 [42], [55], and this may account for the specificity of the CHD5-HDAC2 interaction we observed in neurons and brain.

We fractionated nuclear proteins using size-exclusion chromatography, to ask whether CHD5 protein also existed as monomers. CHD5 migration was approximately megadalton, and little or no CHD5 was detected at the expected size for monomeric or dimeric CHD5 (Figure 4c), suggesting CHD5 was predominantly present in a large complex similar in size to NuRD and SWI/SNF ATP-dependent remodeling enzymes. HDAC2 and p66ß co-migrated with CHD5, consistent with the proteins being present in a NuRD-like complex (Figure 4c). We did not detect smaller complexes, as found for Mi2 proteins in flies.

### CHD5 depletion alters neuronal expression of genes implicated in aging, Alzheimers', and neuronal function

**Figure 5:**
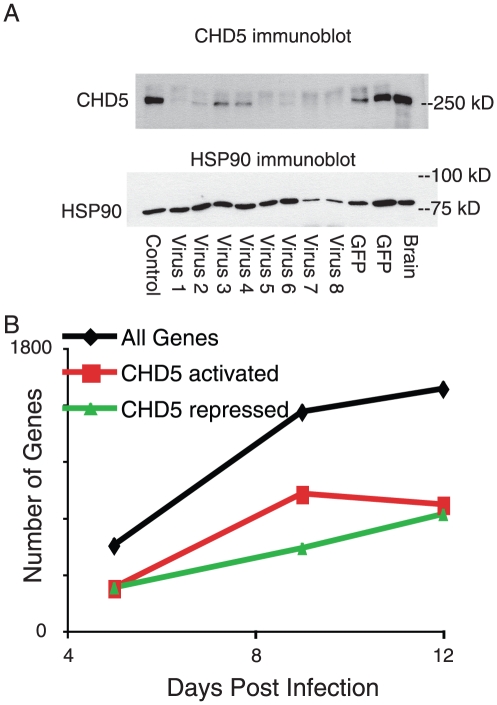
Inhibition of CHD5 protein expression in primary rat neurons. A) Neurons from E18 rats were cultured, infected with the indicated CHD5 lentivirus (1–8) or control lentivirus (Control shRNA, GFP) on day 1, protein was harvested at day 5, analyzed by SDS-PAGE, transferred, and immunoblotted with antisera recognizing CHD5 (HD5A-E day 77) or HSP90 (a loading control). Mouse brain nuclear extract is shown as a positive control. B) Neurons from E18 rats were cultured, infected with CHD5 lentivirus 1 or control lentivirus on day 1, and mRNA was harvested at day 5, 9 and 12. cDNA was synthesized, then quantified using Illumina microarrays. The number of genes that were upregulated (green) and downregulated (red) in a statistically significant manner was counted at days 5, 9, and 12. “All genes” is the sum of genes upregulated and dowregulated by inhibition of CHD5 protein. Microarray data are deposited in GEO (GSE27620).

We hypothesized that CHD5 might regulate neuronal gene expression. To deplete primary neurons of CHD5, we constructed lentiviral vectors to express shRNA targeting CHD5. We identified sequences that reduced the amount of CHD5 protein expressed in primary rat neurons (Figure 5a); shRNA that reduced CHD5 mRNA also reduced CHD5 protein. We identified CHD5-dependent gene expression in primary neurons using microarray analysis (described in detail in Methods S1); primary data were deposited in GEO (GSE27620). We found the expression of several hundred genes was augmented or diminished following depletion of CHD5 protein; approximately similar numbers of genes were affected in each direction (Figure 5b). Pathways from the MSigDB database of the BROAD institute of MIT were queried for regulation by CHD5; these gene sets come from many cell types and sources [56]. At both day 9 and day 12 of differentiation, the most strongly downregulated gene sets were “AGEING_BRAIN_UP”, a gene set identified from aging human brain, [57] and “STEMCELL_NEURAL_UP”; “ALZHEIMERS_DISEASE_UP”, a gene set identified in human brains of Alzheimer's patients [58] was also strongly downregulated, while “ALZHEIMERS_INCIPIENT_UP” [58] was downregulated less strongly (Table S2). The “AGEING_BRAIN_DN” gene set [57] was upregulated at the same times; thus, CHD5 had opposite effects on genes that were upregulated and downregulated in the human brain during aging. At day 5, “RETT_DN” [59] and “NGFPATHWAY” were also upregulated at day 5. To our knowledge, CHD5 had not previously linked to aging or Alzheimer's disease.

Among GO term gene sets, defects in expression of neuronal gene sets were prominent (Table S3). “NEURON FATE COMMITMENT” was significantly upregulated at day 12, “BEHAVIORAL FEAR RESPONSE” was downregulated at days 9 and 12; “NEGATIVE REGULATION OF NEURON DIFFERENTION” was upregulated at days 9 and 12, “SYNAPSE ORGANIZATION AND BIOGENESIS” was upregulated at day 12, “NEURON PROJECTION” was upregulated at days 5 and 9, while “NEURITE DEVELOPMENT”, “FOREBRAIN DEVELOPMENT”, and “DENDRITE” were upregulated at day 9. Cell cycle defects were also evident: “CELL DIVISION” was significantly upregulated at day 12; “REGULATION OF PROGRESSION THROUGH CELL CYCLE” was downregulated days 5, 9, and 12; “CYCLIN DEPENDENT PROTEIN KINASE ACTIVITY” was downregulated at days 5 and 12. CHD5 had not been identified as part of any of these pathways prior to our work.

**Figure 6:**
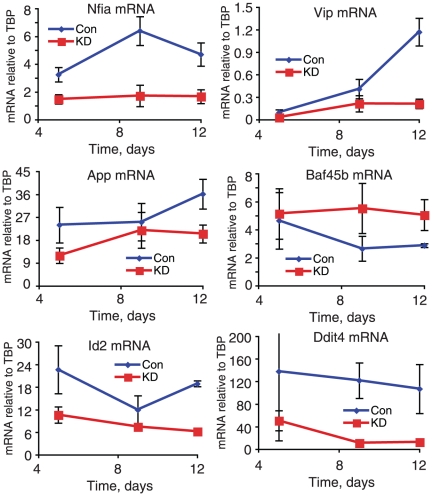
Depletion of CHD5 protein alters gene expression in primary rat neurons. Rat neurons were harvested and plated (D0), infected with CHD5 or control lentivirus 24 hours later (D1) and mRNA was harvested Day 5, 9, and 12. cDNA was synthesized, and quantified using quantitative PCR. mRNA amounts were normalized to the housekeeping gene TBP. “KD” indicates infection with CHD5 lentivirus, “Con” indicates infection with control lentivirus. Symbols indicate average values from 3 plates, while error bars indicate standard deviations. The absolute expression of these genes is quite different, so they are plotted to different scales.

We validated selected expression changes using independent biological samples. Targets were considered validated by us when the initial microarray result (performed as a timecourse, in triplicate) was reproduced in at least 3 independent replicate experiments (also performed as timecourses in triplicate); any target that did not replicate in the experiment performed using a different shRNA reagent was considered a potential off-target result. We tested genes previously implicated in Alzheimer's disease through gene set enrichment analysis, and confirmed that App and ApoE were downregulated by a reduction in CHD5, suggesting CHD5 was activating their expression (Figure 6 and Table S4). CHD5 mRNA was also downregulated by the knockdown, while the highly homologous CHD3 and CHD4 were unaffected (data not shown). We next validated genes with known neuronal function including Vip, Npy, Maoa, Dlx1, Nrxn1, Fmr1, Dlx1, Nefl, and Nefm (Figure 6 and Table S4). Reduction in CHD5 expression reduced the amount of mRNA for these genes. For other neuronal genes (L1cam, Gabrd, Ampd3) CHD5 functioned as a repressor (Table S4). We confirmed CHD5 activation of expression of the transcription factors Nfia and Id2 (Figure 6 and Table S4). Nfia was previously identified as an activator of Ndrg2 expression in brain [60]; we verified Ndrg2 expression was reduced by CHD5 inhibition (Table S4), which may be mediated by loss of Nfia. Finally, we examined chromatin remodeling enzymes and other regulators, confirming expression increased for the SWI/SNF components Baf45b/Neud4, Baf53b/Actl6b and BAF 60a/Smarcd1, the histone methyltransferases Suv39h1 and Suv420h2 and the micro-RNA processing enzyme Dicer (Figure 6 and Table S4). Baf45b and Baf53b are both brain specific components of the SWI/SNF complex varaint nBAF, which is also restricted to brain [36], [38], [39]. The functions of nBAF include development of neurons after neuronal precursor formation and dendrite formation. We found the GO term “DENDRITE” was upregulated at day 9 following inhibition of CHD5, consistent with our finding that CHD inhibition increased expression of the nBAF components Baf45b and Baf53b.

We note that the effects of CHD5 depletion are most prominent at different times for different genes. We do not yet understand why this is. For genes such as Id2 and Nfia, CHD5 alters gene expression at the earliest time tested, while for other genes such as Vip and Baf45b, the effect of CHD5 depletion is not detected until later. We hypothesize that in these cases, CHD5 might be working with a pathway that is turned on after several days of culture. For example, CHD5 might activate Vip through a transcription factor that is repressed or inactive at day 5, and expressed/activated later, in which case CHD5 would have no detectable effect until the transcription factor recruiting CHD5 was functional.

### CHD5 binds target genes, suggesting direct regulation

**Figure 7:**
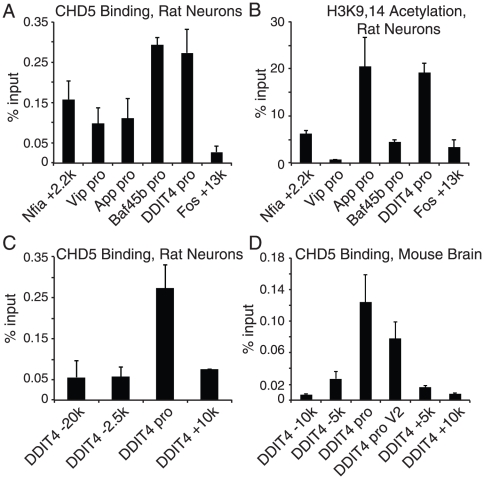
CHD5 protein binds to target genes in primary rat neurons and mouse brain. Neurons from rats were cultured; neurons and brain were crosslinked with formaldehyde, and analyzed by ChIP-Q-PCR with the indicated antibodies (for CHD5, antisera HD5A-A day 77 was used) as described in the methods. Fos +13.4k locus is one measure of background for CHD5 ChIP; we have not found CHD5 binding to this region. Another measure of background is ChIP with control IgG; the mean+standard deviation for all loci was approximately equal to 0.05 in rat primary neurons, similar to the signal we see at Fos +13.4k, and 0.02 in mouse brain. The gene name and the distance from the TSS are listed below. Data are shown as the averages and standard deviations of three experiments performed with independent preparations of primary rat neurons and mouse brains. A) CHD5 binding, primary rat neurons. B) H3K9,14 acetylation, (a mark associated with some active chromatin), primary rat neurons. C) CHD5 binding, primary rat neurons. D) CHD5 binding, mouse brain.

As noted above, changes in gene expression caused by reduction in CHD5 protein could be the direct result of CHD5 function on these target genes. Alternatively, CHD5 function could be indirect, for example CHD5 might regulate expression of a regulator of a target gene. To distinguish these possibilities, we performed ChIP for CHD5 on cultured rat neurons. We found CHD5 binding to the CHD5 targets Nfia, Vip, App, Baf45b, and Ddit4 (Figure 7a). CHD5 binding was 2 to 10 fold greater than control IP at these target loci, while CHD5 binding was at background (similar to control IP) at other loci such as downstream of the Fos promoter (+13.4k). There was no simple correlation between CHD5 binding and H3K9,14 acetylation or H3K27me3 (Figure 7b and data not shown), suggesting it was unlikely these positive and negative marks served to recruit CHD5. We investigated sequences flanking the DDIT4 promoter to test our ChIP specificity; we found little if any CHD5 signal at several nearby loci (Figure 7c). We extended our ChIP to mouse brain; using an organ obtained from an animal, rather than cultured cells, we found CHD5 binding to the CHD5 promoter using 2 different primer sets (Figure 7d). Again, CHD5 signal was absent at flanking regions, confirming the specificity of our CHD5 ChIP in a second species.

CHD5 has been identified as a tumor suppressor for neuroblastoma. However, little is known about the function of CHD5. We found CHD5 was a nuclear protein predominantly found in neurons. We found CHD5 was in a megadalton, NuRD-like complex containing HDAC2, p66ß, MTA3, RbAb46 and MBD3. CHD5 was present early in development and in adult brain, and CHD5 was expressed in several brain regions, implying CHD5 may play a broad role in brain development or function. CHD5 appears to regulate gene expression, as inhibition of CHD5 protein expression alters mRNA amounts of many genes. To our knowledge, this is the first report of gene regulation by CHD5 in neurons. A previous report of gene regulation by CHD5 was performed in fibroblasts, and did not determine whether CHD5 bound the target genes [4]. The regulation we observed may be direct in some cases, as CHD5 binding was found at a number of these target genes at promoters and distal sites. Using microarrays and pathway analysis, we found CHD5 regulated genes that were previously found to change expression in response to aging or Alzheimer's disease, suggesting CHD5 may play a broader role in human disease than currently recognized.

## Supporting Information

Figure S1 **CHD5 motifs and sequence homology.** A) The human protein sequences of the indicated ATPases were aligned using MacVector (Accelrys Inc.). The Mi2, SWI/SNF, ISWI, CHD1/2, and CHD6-9 subfamilies are indicated. CHD5 and Mi2 subfamily are marked in red. B) Motifs from representative ATPase family members were identified using PROSITE [61]. The ATPases and domains are drawn to the same scale; increasing length corresponds to more amino acids. The N terminus of each protein is at the left. PhD domains are in red, chromo domains are in blue, ATPase domains are in green, and other domains not found in the Mi2 subfamily are shown in black. Ab1 indicates region targeted by antisera HD5A-E, and Ab2 indicates region targeted by antisera HD5A-A.
(EPS)

Figure S2 **CHD5 is capable of binding HDAC1.** HeLa cells were transfected with human CHD5 cDNA (shown) or control (not shown). Extracts were immunoprecipitated with the indicated antisera, analyzed by SDS-PAGE, transferred, and detecting by immunoblotting with the indicated antisera. Note that CHD5 IP does not enrich for CHD4, suggesting these proteins are not associated, and CHD5 antisera does not cross-react with CHD4.
(EPS)

Table S1 **Peptide sequence from CHD5 and associated proteins.** Table S1a- CHD5 Peptides. Table S1b- HDAC2 Peptides. Table S1c- Gatad2b/p66ß Peptides. Table S1d- MTA3 peptides.
(DOCX)

Table S2 **Gene sets altered in a statistically significant manner by CHD5 inhibition.** The columns “Pathway Name” and “Annotation” list gene sets from the molecular signature database. Z scores for changes in the gene set are listed in the following columns. “D12KD_D12C” compares gene set expression after treatment with CHD5 shRNA lentivirus to contol, at day 12. Negative Z scores indicate expression of the gene sets are reduced, and thus CHD5 is formally an activator, while positive Z scores indicate expression of the gene sets are increased, and thus CHD5 formally represses them. “D9KD_D9C” and “D5KD_D5C” measure the effect of CHD5 depletion at earlier times. “D12C_D5C” and “D9C_D5C” compare the change in gene set expression in control cells at day 12 and day 9 relative to day 5.
(XLS)

Table S3 **GO term gene sets altered in a statistically significant manner by CHD5 inhibition.** The columns “Gene Ontology Term” and “Annotation” list gene sets from the molecular signature database. Z scores for changes in the gene set are listed in the following columns. “D12KD_D12C” compares gene set expression after treatment with CHD5 shRNA lentivirus to contol, at day 12. Negative Z scores indicate expression of the gene sets are reduced, and thus CHD5 is formally an activator, while positive Z scores indicate expression of the gene sets are increased, and thus CHD5 formally represses them. “D9KD_D9C” and “D5KD_D5C” measure the effect of CHD5 depletion at earlier times. “D12C_D5C” and “D9C_D5C” compare the change in gene set expression in control cells at day 12 and day 9 relative to day 5.
(XLS)

Table S4 **Summary of CHD5 targets validated by Q-RT-PCR.** The column “Target of CHD5” lists genes identified as CHD5 targets using microarray analysis. The column “CHD5 Function” lists the result validated in at least 3 independent experiments. “Activator” indicates expression was reduced following CHD5 depletion for at least one time window, “Repressor” indicates expression was increased following depletion, while Activator/Repressor indicates both, at different times. “CHD5 Binding” indicates measurement by ChIP; Yes indicates binding above control IP and control locus. “Region(s) indicate where binding was (or was not) observed. “Gene Sets” indicates, for the gen in that row, the gene sets from Table S2 this gene is found.
(XLS)

Table S5 **Sequences.** 5A) Primers for measuring mRNA amounts using Q-RT-PCR. 5B) Mouse primers. 5C) Rat primers. 5D) Primers for detecting proteins in rat chromatin using ChIP. 5E) Sequences and names for pLKO.1 lentivirus shRNA constructs. 5F) Primers for expressing hCHD5 fragments to raise antisera.
(DOCX)

Methods S1 **Production of CHD5 antisera, characterization of CHD5 antisera specificity, isolation of mouse brain nuclear extracts, measurement of mRNA from mouse and human organs, Illumina BeadChip analysis, and Pathway analysis are described.** Additional references are provided.
(DOCX)
