# Supplementary material for: CHD5, a Brain-Specific Paralog of Mi2 Chromatin Remodeling Enzymes, Regulates Expression of Neuronal Genes
Source: PLoS One. 2011 Sep 13;6(9):e24515. doi: 10.1371/journal.pone.0024515 (PMC3172237; doi:10.1371/journal.pone.0024515)
Supplement: Table S1 — Peptide sequence from CHD5 and associated proteins. Table S1a- CHD5 Peptides. Table S1b- HDAC2 Peptides. Table S1c- Gatad2b/p66ß Peptides. Table S1d- MTA3 peptides. (DOCX) [file pone.0024515.s003.docx]

Table S1a- CHD5 Peptides:

Summary:

CHD5- 10 peptides total

CHD5 only- 4 peptides

CHD5 and (CHD4 and/or CHD3)- 6 peptides

(CHD4 and/or CHD3), not CHD5- none

Run 1

VGGNIEVLGFNAR (CHD5, CHD4)

Run 2

GPYLVSAPLSTIINWER (CHD5)

TVQTIVFLYSLYK (CHD5)

ENEFSFEDNAIR (CHD5, CHD4)

VGGNIEVLGFNAR (CHD5, CHD4)

LLEQALVIEEQLR (CHD5, CHD4, CHD3)

Run 3

AAYLNMTQDPNHPAMALNAR (CHD5)

LLDRNQDATDDTELQNMNEYLSSFK (CHD5)

HHYEQQQEDLAR (CHD5, CHD4, CHD3)

FSWAQGTDTILADEMGLGK (CHD5, CHD4, CHD3)

Table S1b- HDAC2 Peptides:

Summary

HDAC2- 9 total

HDAC2 only- 2 peptides

HDAC2 and HDAC1- 7 peptides

HDAC1, not HDAC2- none

Run 1

SIRPDNMSEYSK(HDAC2, HDAC1)

YGEYFPGTGDLR (HDAC2, HDAC1)

DGIDDESYGQIFKPIISK (HDAC2, HDAC1)

MTHNLLLNYGLYR (HDAC2, HDAC1)

Run 2

LHISPSNMTNQNTPEYMEK (HDAC2)

YHSDEYIK (HDAC2)

SIRPDNMSEYSK (HDAC2, HDAC1)

YGEYFPGTGDLR (HDAC2, HDAC1)

MTHNLLLNYGLYRK (HDAC2, HDAC1)

Table S1c- Gatad2b/p66β Peptides:

Summary

Gatad2b/p66β- 18 peptides total

Gatad2b/p66β only- 18 peptides

Gatad2a/p66α, exact match- none

Run 1

ALQQEQEIEQR (Gatad2b/p66β)

VIAPNPAQLQGQR (Gatad2b/p66β)

SLDPADERDDVLAK (Gatad2b/p66β)

LQQQAALSPTTAPAVSSVSK (Gatad2b/p66β)

VSSPLPSPSAMSDAANSQAAAK (Gatad2b/p66β)

DLANLEVPHELPTK (Gatad2b/p66β)

Run 2

LQQQAALSPTTAPAVSSVSK (Gatad2b/p66β)

TPVVQNAASIVQPSPAHVGQQGLSK (Gatad2b/p66β)

Run 3

TAGRPGKENINDEPVDMSAR (Gatad2b/p66β)

SLDPADERDDVLAK (Gatad2b/p66β)

QLRDELRLEEAR (Gatad2b/p66β)

VSSPLPSPSAMSDAANSQAAAK (Gatad2b/p66β)

TPVVQNAASIVQPSPAHVGQQGLSK (Gatad2b/p66β)

SATNTTLPHMLMSQR (Gatad2b/p66β)

DLANLEVPHELPTKQDGSGVK (Gatad2b/p66β)

LTPSPDIIVLSDNEASSPR (Gatad2b/p66β)

TTSSAIYMNLASHIQPGTVNR (Gatad2b/p66β)

ALQQEQEIEQR (Gatad2b/p66β)

Table S1d- MTA3 peptides:

Summary

MTA3- 6 peptides total

MTA3 only- 4 peptides

MTA3 and MTA1- 2 peptides

MTA1, MTA2, not MTA3- none

Run 1

ETEEESETPVEADLTEK (MTA3)

AGTVNGAVGTQFQPQSALLGR (MTA3)

VGDYVYFENSSSNPYLIR (MTA3, MTA1)

DITLFHAMDTLYR (MTA3)

Run 2

AGTVNGAVGTQFQPQSALLGR (MTA3)

VGDYVYFENSSSNPYLIR (MTA3, MTA1)
