# Supplementary material for: CHD5, a Brain-Specific Paralog of Mi2 Chromatin Remodeling Enzymes, Regulates Expression of Neuronal Genes
Source: PLoS One. 2011 Sep 13;6(9):e24515. doi: 10.1371/journal.pone.0024515 (PMC3172237; doi:10.1371/journal.pone.0024515)
Supplement: Table S5 — Sequences. 5A) Primers for measuring mRNA amounts using Q-RT-PCR. 5B) Mouse primers. 5C) Rat primers. 5D) Primers for detecting proteins in rat chromatin using ChIP. 5E) Sequences and names for pLKO.1 lentivirus shRNA constructs. 5F) Primers for expressing hCHD5 fragments to raise antisera. (DOCX) [file pone.0024515.s007.docx]

Table S5. Sequences.

5A) Primers for measuring mRNA amounts using Q-RT-PCR.

Human primers

| Gene | Name | Primer 1 | Primer 2 |
| --- | --- | --- | --- |
| CHD5 | MP 1249 | CCTGAAAAAGTGCTGCAACCA | TTGACCAGGGAGCTTCCATC |
| TBP | MP 984 | GCCCGAAACGCCGAATAT | CGTGGCTCTCTTATCCTCATGA |

5B) Mouse primers

| Gene | Name | Primer 1 | Primer 2 |
| --- | --- | --- | --- |
| Chd5 | MP 1049 | GCCCGCGCTGTACATGTC | CAGTGTAGGATCCGCTGAACTTT |
| Snf2H | MP 620 | TGTATTACAGGAAAACTATTGGGTATAAGGTA | TTAGGTAGATCAGGACTCCGAGGTA |
| Brg1 | MP 670 | TCAACGGGCCTTTCCTCA | CCCAGTTTGACAGTGTCGAGAG |
| Brm | MP 777 | CGCCATCATTGATACTGTGATAAAC | TGGAAGGTAACTGAATGAAGACTTCA |
| Chd1 | MP 1107 | TCGCCCCTGCCTTCAGA | GAGCATCGGACACTACAGACTTTTT |
| Chd2 | MP 1108 | TCCTGGCCGAGTACCTGACTAT | CATCCAGGCGCTGGAAAG |
| Chd3 | MP 1109 | GGAGGACTACCACACACTTACCAATT | GACATCGGGATCTTAGGATTCTTC |
| Chd4 | MP 1110 | CTCCTTTGAAGACAATGCCATTC | ATGGTGATCAACTCATAGGATGTCA |
| Chd7 | MP 1050 | CAGAGATTGAGGATGACCTTTTTAATC | GTGCTACGTGCAAAGTCCATTATC |
| Tbp | MP 935 | CTTCGTGCAAGAAATGCTGAATAT | TGTCCGTGGCTCTCTTATTCTCA |

5C) Rat primers

| Gene | Name | Primer 1 | Primer 2 |
| --- | --- | --- | --- |
| Chd5 | MP 1539 | AGAAGGGTTCCTCGAGCGA | TGTCCAAATCTGAGTCCTCCG |
| Tbp | MP 1528 | CTTCGTGCCAGAAATGCTGA | GATGACTGCAGCAAACCGC |
| Ndrg2 | MP 1765 | GGGCACCTGTGTTTCCTCTG | CAAGCTGGTCCTGAGATGGG |
| Nfia | MP 1764 | AGCAGCCTGGGCCTTATTTC | CCTGAGGGTGGTAACGGATG |
| ID2 | MP 1768 | GAAAGCCTTCAGTCCGGTGA | TGGTCCGACAGGCTGTTTTT |
| Fos | MP 1542 | CCTTCTCCAGCATGGGCTC | GATCTGCGCAAAAGTCCTGTG |
| Baf45b/Neud4 | MP 1766 | CCCGAGGCACAGAGGAAAC | GACAGTGCCATCTGGTGCTTT |
| Baf 60a | MP 1776 | GGAAGCTGCGGATTTTCATTT | CATCCGACTTAGCCGGATTG |
| Suv39h1 | MP 1783 | GAGTTGGTGAGGGCATCACC | GGCACTCACAGCCAACAGC |
| Suv420h2 | MP 1782 | GCCACCAGCCTTGTTCTTGA | CATCTTATGGGTGCGGAAGC |
| Satb1 | MP 1795 | GTTTTACAAACTGCTTGGCGG | ACCCAACAATCCTTGACGCT |
| Dicer | MP 1796 | GTTTAGCCCAGCGGAGAGAA | TCCACGGTGACTCTGACCTTC |
| APOE | MP 1762 | CAGACGCTTTCTGACCAGGTC | TGTGACTTGGGAGCTCTGCA |
| APP | MP 1709 | CTACGAGCGCATGAACCAGTC | ACGGCAGGGACGTTGTAGAG |
| VIP | MP 1789 | GAAAGACCCAAGGAGGCACC | GGAACTGAGGCTTGCTTCTGG |
| NPY | MP 1700 | CCCGCCATGATGCTAGGTAA | GAGGGTCAGTCCACACAGCC |
| GFAP | MP 1533 | GGAACATCGTGGTAAAGACGG | CCTTAATGACCTCGCCATCC |
| Grin1/NMDAr1 | MP 1772 | GGCTTCACAGAAGTGCGATCT | GAGCGGAAGAACAGCTCACC |
| Adora2 | MP 1692 | GGCTCAGGTCCCATGAGAGA | GGTTCAGGTCCATGGCTTGA |
| NEFM | MP 1697 | CACATCACCGTAGAGCGCAA | CGCCGTGGAGATGTCTGTCT |
| NEFL | MP 1698 | GAAGGCGAAGAAACCAGGCT | GCTGGTTATGCTACCCACGC |
| FMR1 | MP 1541 | TCAGCTGGTAATTTTGTCCATCA | ATGTGGGCTCGCTTTGAGG |
| Nrxn1 | MP 1857 | TGACCGACCCAGTACACGAG | ACAGTGCTAAACCCGATGGC |
| DDIT4 | MP 1865 | TGGCATCAGTTCGCTCACC | GATTGCGGACAAGAGCGATC |
| MAOA | MP 1860 | GTAGTCGTGATTGGAGGCGG | CAACAGTTTGGCAGCAGCC |
| Ampd3 | MP 1866 | AACGCTTGCTGGTCGGTTTA | GGAATTGGCGAGGCATCTC |
| Dlx1 | MP 1864 | ATCCCCGGTGATTATGCAAG | CATGAGCTGGTGGAGCTGATT |
| L1CAM | MP 1858 | ACACGAGGCACCCTATTCTGG | AAGCTGTTGTTGCCTTCGATG |
| GABRD | MP 1862 | GTGGCTCCCTGACACCTTCA | TCATGGAACCAGGCAGACTTG |
| FGFr3 | MP 1863 | CGACCGGAGCGTATGGATAA | AGTGTTTGCAGCTGGCACAG |
| Add3 | MP 1867 | GCTGGTTCTGTCTGAGCGTG | AGTGAGGTCTACGGCGACGT |
| Chd3 | MP 1780 | GTTCATGCGCCATCTGTGC | CGCAAAGGTTTCAGAGCCAT |
| Chd4 | MP 1781 | TGCTTCTTGCTTTCCACTCGA | AGTGGCCAGATTGATCCCAA |
| Baf53b/Actl6b | MP 1767 | GTCATGTCGCCCCTCAAGAA | TCGGAAGCACTCCCAGTCC |

5D) Primers for detecting proteins in rat chromatin using ChIP.

| Gene | Location | Name | Primer 1 | Primer 2 |
| --- | --- | --- | --- | --- |
| Ndrg2 | +1.8k | MP 1890 | TCGCTGCCACCTTCACTTG | GAGCCTGAACTAGGGCGGAC |
| Nfia | +2.2k | MP 1906 | CTGCTGAAAGGATCGCAACTT | TGCCTGGTGTGCTTGAAAGA |
| Nfia | +34k | MP 1907 | TGTGGGAACATTCACTTGAGAAGA | TTGGCCATAAAGCTGCAGAA |
| Nfia | +102k | MP 1891 | GCTTCTGTCACCTCCCCCAT | AAGCGGGAAAGTGACTGGC |
| Id2 | -96k | MP 1887 | GAGCCTACCCACAGCATCCTC | TCTCGTTCCTGGAACCGTCT |
| Id2 | -29k | MP 1903 | CCATGTCAATCCCCGAGTAGA | CATTCAGCCCACTGCTGTGT |
| Id2 | -3k | MP 1888 | TGCAGCCTCTGCACGTCTTA | TCTTCCTTCTTCCCCCAAATC |
| Fos | -1.5k | MP 1496 | AGGGCAGGTTTGTGGTTCTG | AGCGGATGGATCTTCAGGG |
| Baf45b | Pro | MP 1755 | CCTGATTGGTTGAGATCCTCG | CCATCCATTCATTCCCGG |
| Baf45b | +7.3k | MP 1900 | CAGTCGAGCTTCCGGATGTT | AGCTCTGCAGTGGGACCCTA |
| Baf60a | Pro | MP 1878 | CCCGGCACTTCTTTATCAGC | GCCAGGAGGGAGCACCATA |
| Satb1 | -75k | MP 1894 | AGGAAGGACGCACATCGTG | GGCGGAAGTCACCACAACTT |
| Dicer | Pro | MP 1882 | TGAAAGCCGTCCTGTGATTG | CGGGATTAACCTTTCACCTCC |
| Apoe | Pro | MP 1855 | CAACTCCTTAGCCTCCGGG | AATTGGACAGGTCTGGGATCC |
| App | -154k (v) | MP 1899 | TACAGATGGCTAGCAGCCCC | GGACACCTCTGCAGCTGGTC |
| App | Pro | MP 1856 | CAGCATCGTGATCCTGCGT | TCTTCCACTTGCACACGGAG |
| Vip | Pro | MP 1897 | CTGTGACGTCTTTCGAGGAGC | GGCTTGTGCTTAGGGCTGAG |
| Npy | -96k | MP 1910 | AGGACGCTTCTGAGAGTGGG | CCCTTCACATGAGCATTGCA |
| Npy | Pro | MP 1851 | AAAAGCCCGTTGGCGAC | AGGAATGAGCTCCACCGGT |
| Gfap | Pro | MP 1885 | CTCCATCTTGCCCTGCTTCT | CAATAAAGGCCCTGACATCCC |
| Grin1 | Pro | MP 1886 | GAGGTGCTGAAGCGTGTTTG | TGTCCTTTCCGCCTTTTCC |
| Adora2 | +0.5k | MP 1874 | ACCGGAAGGTGCCTAAGGG | CCTGCCTTACTCGAACCCC |
| Adora2 | +3.3k | MP 1875 | CCTCCAGAGAGCTGCAGAGAA | GACAGGATGTGGGCAGGAAG |
| Nefm | Exon 1 | MP 1490 | GCCTTCTCGTGATTCACCATC | ACCAGGAGATCCGAGAGCTG |
| Nefl | -40.5k (v) | MP 1904 | CTTGGTCCTCTCTCCAGGCC | CCTCGGTTCAATTTGCTGGT |
| Nefl | Pro | MP 1746 | AATAGAGGTGGCAGGACAGCC | GAAAGGGAAGGATGGATGGC |
| Fmr1 | Pro | MP 1518 | CTCAGTCAGTCTTGCGCTGG | CCCCTCACCGGAAGTGAAA |
| Nrxn1 | +242k | Mp 1911 | GGGAAGCCACACAGGAACC | CTCATTCTTCCCCCAGCAAG |
| Nrxn1 | +1253k | MP 1913 | CCACCCATTGTCTCCACTCC | GATGTCCAGATGGGATTGGC |
| Ddit4 | -20k (v) | MP 1879 | CCGCAGGGTAAGTTCAGAGC | CCTGTGGGTGAGCTGAGAACA |
| Ddit4 | -11k | MP 1880 | TGGGTGGGAGTACTGCACTG | CTCAAATGTGGGAGGAGGGA |
| Ddit4 | -2.5k | MP 2037 | GGAGACACAGTTAATGACAGCTGC | CCAGAAGCAAACACGGCACT |
| Ddit4 | Pro | MP 1881 | GGACACTCACCAGCTAGCGC | AGATCGCTCTTGTCCGCAAT |
| Ddit4 | +10k | MP 2049 | TTGGTTCTCTGTGCCCTTGTT | CACCCAGACCAGAAGCTTGG |
| Maoa | Pro | MP 1889 | AACATGTGGCCTGCGAGATT | CACCCAAACCATGACGGATC |
| Dlx1 | -12k | MP 1902 | GGCGCTCTTGCATTTTAACAA | TCTCTGCCCCCTTCGACTG |
| Dlx1 | Pro | MP 1883 | TGAGCTAGGCACAGGACGCT | ACAACCCAGGTAGCCTGCAG |
| Fgfr3 | Pro | MP 1884 | AGGCTCGGAAGTAACTCCAGC | TGCGTGCTAGTGTTCTGCGT |

5E) sequences and names for pLKO.1 lentivirus shRNA constructs.

| Name | Primer |
| --- | --- |
| MP 1685a | AATTCAAGAAGTACTACAAGTTCATCCTCGAGGATGAACTTGTAGTACTTCTTTTTTTG |
| MP 1685b | TAATTCAAAAAAAGAAGTACTACAAGTTCATCCTCGAGGATGAACTTGTAGTACTTCTT |
| MP 1686a | AATTCAAGAAGAAAGAGGGGAGCAATCTCGAGATTGCTCCCCTCTTTCTTCTTTTTTTG |
| MP 1686b | TAATTCAAAAAAAGAAGAAAGAGGGGAGCAATCTCGAGATTGCTCCCCTCTTTCTTCTT |
| MP 1687a | AATTCAACATGAACGAGTACCTGAGCCTCGAGGCTCAGGTACTCGTTCATGTTTTTTTG |
| MP 1687b | TAATTCAAAAAAACATGAACGAGTACCTGAGCCTCGAGGCTCAGGTACTCGTTCATGTT |
| MP 1688a | AATTCGGGCTACAAGTATGAGCGGATCTCGAGATCCGCTCATACTTGTAGCCCTTTTTG |
| MP 1688b | TAATTCAAAAAGGGCTACAAGTATGAGCGGATCTCGAGATCCGCTCATACTTGTAGCCC |
| MP 1689a | AATTCTAGGGTCTTGAATAGCTACAACTCGAGTTGTAGCTATTCAAGACCCTATTTTTG |
| MP 1689b | TAATTCAAAAATAGGGTCTTGAATAGCTACAACTCGAGTTGTAGCTATTCAAGACCCTA |
| MP 1690a | AATTCTAGGAAGAAGGTGCAGGAGTTCTCGAGAACTCCTGCACCTTCTTCCTATTTTTG |
| MP 1690b | TAATTCAAAAATAGGAAGAAGGTGCAGGAGTTCTCGAGAACTCCTGCACCTTCTTCCTA |
| MP 1691a | AATTCGAGCGAGAAACGGAAGAACAACTCGAGTTGTTCTTCCGTTTCTCGCTCTTTTTG |
| MP 1691b | TAATTCAAAAAGAGCGAGAAACGGAAGAACAACTCGAGTTGTTCTTCCGTTTCTCGCTC |

5F) Primers for expressing hCHD5 fragments to raise antisera

| Name | Fragment | Primer |
| --- | --- | --- |
| MP 1051a | Chd5 1-84 | CGGGATCCATGCGGGGCCCAGTGGGCACCGAG |
| MP 1051b | Chd5 1-84 | CCGCTCGAGTTATCACTACTCTTCCAGATCCTCTTCATTCTC |
| MP 1052a | Chd5 251-336 | CGGGATCCGGAGTGAGGAAGAAGATCAAAGGC |
| MP 1052b | Chd5 251-336 | GACGTCGACTTATCACTAGTCACCATCATCAATCCTCTTCTT |

5G) Primers for detecting proteins in rat chromatin using ChIP

| Gene | Location | Name | Primer 1 | Primer 2 |
| --- | --- | --- | --- | --- |
| Ddit4 | -10k | MP 2051 | TGTGTAGCCCTGGGTGTCCT | CGAGGCCAGTCTGGTCTACAG |
| Ddit4 | -5k | MP 2050 | AGTTGGAGCACAGCATCCCT | TGCTGAGGTTGATTGGTTGC |
| Ddit4 | Pro | MP 1881 | GGACACTCACCAGCTAGCGC | AGATCGCTCTTGTCCGCAAT |
| Ddit4 | Pro V2 | MP 2054 | TCAGCCAATAGGCACCGG | TCACCAGGCAGGAGAGAACG |
| Ddit4 | +5k | MP 2052 | CCTCTGCACTTTCTCCTCACG | GGCTCAGGGAACACTAGGGC |
| Ddit4 | +10k | MP 2053 | CCGACCCCATTACAGATGGTT | TCAATTCCCAGCGACCACAT |
