# Supplementary material for: CHD5, a Brain-Specific Paralog of Mi2 Chromatin Remodeling Enzymes, Regulates Expression of Neuronal Genes
Source: PLoS One. 2011 Sep 13;6(9):e24515. doi: 10.1371/journal.pone.0024515 (PMC3172237; doi:10.1371/journal.pone.0024515)
Supplement: Methods S1 — Production of CHD5 antisera, characterization of CHD5 antisera specificity, isolation of mouse brain nuclear extracts, measurement of mRNA from mouse and human organs, Illumina BeadChip analysis, and Pathway analysis are described. Additional references are provided. (DOCX) [file pone.0024515.s008.docx]

**CHD5, A Brain-Specific Paralog of Mi2 Chromatin Remodeling Enzymes, Regulates Expression Of Neuronal Genes.**

Rebecca Casaday Potts^1,4^, Peisu Zhang^2^, Andrea L. Wurster^1^, Patricia Precht^1^, Mohamed R. Mughal^2^, William H. Wood III^3^, Yonqing Zhang^3^, Kevin G. Becker^3^, Mark P. Mattson^2^ and Michael J. Pazin^1,5^

Laboratory of Molecular Biology and Immunology^1^, Laboratory of Neuroscience^2^, Research Resources Branch^3^

National Institute on Aging Intramural Research Program, National Institutes of Health

^4^ present address:

U.S. Pharmacopeia

Rockville, Maryland 20852

^5^ Corresponding Author, present address:

NHGRI, NIH

5635 Fishers Lane

Bethesda, MD 20892

Phone: 301.443.6811

Fax: 301.480.2770

e-mail: [pazinm@mail.nih.gov](mailto:pazinm@mail.nih.gov)

**Supplementary material**

Contents:

Supplementary methods

Supplementary reference

**SUPPLEMENTARY METHODS**

**CHD5 Antisera**. CHD5 antisera were raised against human CHD5 sequence as modified from [[1](#_ENREF_1)]. We chose regions of CHD5 that were hydrophillic with high surface probability (identified using MacVector, Accelrys Inc.), conserved among species (human, mouse, rat), but not homologous to the closely related Mi2 proteins CHD3 and CHD4. The selected regions were CHD5 amino acids 1-84 and 251-336, with respect to the human sequence, using the primers in Table S5F. Phusion polymerase (NEB) was used to amplify regions of CHD5 from human brain cDNA (human total RNA, Ambion 6000). CHD5 fragment 1-84 was cloned into pHM6-SPIRC vector BamHI/XhoI, CHD5 fragment 251-336 was cloned into pMALC2X vector BamHI/SalI. Maltose-Binding Protein fusion proteins were expressed in Rosetta 2 cells (Novagen); induced with 0.1M IPTG for 3 hours. MBP-fusion proteins were purified using Amylose resin (NEB) and eluted with 10mM maltose, according to the manufacturer’s instructions, and dialyzed against PBS. Josmann Antibody Company immunized rabbits with the purified fusion proteins. Antisera were screened by expressing the CHD5 fragments in 293 cells, which did not contain endogenous CHD5 protein. Antisera HD5A-A day 77 (immunogen: CHD5 amino acids 1-84) was used for ChIP, IP and immunofluoresence. Antisera HD5A-E (immunogen: CHD5 251-336) was used for immunoblotting.

**Antibody specificity**. We demonstrate 1) our antisera do not produce a signal when CHD5 is absent, thus the background is low; 2) our antisera do produce a signal when CHD5 is present, thus they recognize CHD5; 3) our antisera do not recognize the very similar, ubiquitous proteins CHD4 and CHD3, thus they are specific. Antisera HD5A-A day 77 (immunogen: CHD5 amino acids 1-84) was used for ChIP, IP and immunofluoresence. No signal was detected by immunofluorescence using the cell line SH-SY5Y, a cell with a neuronal phenotype but lacking CHD5 expression (Fig 2B); essentially the same antibody control was published previously [[2](#_ENREF_2)]. After transfection with CHD5 cDNA, robust signal was detected (Fig 2B). Staining of brain sections and primary neuronal cultures was nuclear and brain specific (Fig 3); rare glial cells in the primary neuronal cultures were unstained. This antisera immunopurified an endogenous protein of the correct size, with high purity from mouse brain (Fig 4A). Protein sequencing of the proteins purified from mouse brain revealed CHD5 was present (Table S1A), but the paralogs CHD3 and CHD4 were not purified by this antisera (Table S1A), though they are expressed at similar levels in brain (Fig 1B). Protein immunopurified with this antisera was recognized in immunoblots by CHD5- specific antisera (Fig 4B, S2).

Antisera HD5A-E day 77 (immunogen: CHD5 251-336) was used for immunoblotting. Immunblotting revealed this antisera reacted with a protein of the expected size (250 kD) in expected cell types, but not in non-expressing cells (including SH-SY5Y), even though expression of the paralogs CHD4 and CHD3 was robust (Fig 2A, 5A, and data not shown). Knockdown of CHD5 reduced CHD5 mRNA and CHD5 protein detected in primary neurons (Fig 5A). Immunobloting of immunoprecipitates of CHD5 or proteins associated with CHD5 revealed a protein of the expected size (Fig 4B, S2). Immunoblotting of size-fractionated nuclear extracts revealed a 250 kD CHD5 band in a megadalton complex, similar to what has been observed for the paralog CHD4.

**Mouse Brain Nuclear Extracts**. Fresh brains from mice were used, as frozen tissue appear to have degraded remodeling ATPase proteins. Fresh brains were harvested from mice, placed in PBS in Petri dishes on ice, rinsed, transferred to another Petri dish, and minced with a razor blade. The minced brain was transferred to a Dounce using Buffer A (10mM HEPES, pH 7.9, 1.5 mM MgCl_2_, 10mM KCl) +PMSF, Aprotinin, Leupeptin. Buffer A + protease inhibitors was added to about 5x the volume of the settled sample. After incubation for 10 minutes, 4°C, the sample was Dounce homogenized with the loose pestle for 10 strokes. Nuclei were collected by centrifugation (5 minutes, 400xg, 4°C), resuspended and washed with 10x the pellet volume Buffer A+ protease inhibitors. Nuclei were washed 2 more times as above, except the brake was not used for the last centrifugation. The nuclei were resuspended with one packed nuclear volume of Buffer C (20mM HEPES, pH 7.0, 25% glycerol, 0.42M NaCl, 1.5mM MgCl_2_, 0.2mM EDTA) + PMSF, Aprotinin, Leupeptin. (Resuspend by pipet; observe the nuclei to make sure they have not become viscous as this an indication that the nuclei have lysed.) Nuclei were extracted 30 minutes at 4°C, on a rotator. Extract was separated by centrifugation at 15,000xg for 15 minutes at 4°C. The pellet was extracted a second time, and the extracts were pooled. Nuclear extract was flash-frozen in liquid nitrogen, and stored at -80˚C. Nuclear extract was thawed on ice and clarified by ultracentrifuge 100,000xg (34,000 RPM) 4˚C twice for 10 minutes using TL55 swinging bucket rotor and thick-walled tubes.

**CHD5 mRNA in measurement from mouse and human organs**. cDNA was synthesized from RNA of human and mouse tissues (Human Total RNA, Ambion 6000 or Mouse Total RNA, Ambion 7800) using Iscript (BioRad). The cDNA was diluted with water, and real time PCR was used to determine levels of CHD5 expression compared to a housekeeping gene. Primers are listed in Tables S5A and S5B.

**Illunima oligonucleotide Microarray analysis of CHD5-regulated genes.** Transcriptional profiling was determined using Illumina Sentrix BeadChips. Total RNA was used to generate biotin-labeled cRNA using the Illumina TotalPrep RNA Amplification Kit. In short, 0.5ug of total RNA was first converted into single-stranded cDNA with reverse transcriptase using an oligo-dT primer containing the T7 RNA polymerase promoter site and then copied to produce double-stranded cDNA molecules. The double stranded cDNA was cleaned and concentrated with the supplied columns and used in an overnight in-vitro transcription reaction where single-stranded RNA (cRNA) was generated incorporating biotin-16-UTP. A total of 0.75ug of biotin-labeled cRNA was hybridized at 58 degrees C for 16 hours to Illumina's Sentrix Rat Ref-12 Expression BeadChips (Illumina, San Diego, CA). Each BeadChip has ~22,000 well-annotated RefSeq transcripts with approximately 30-fold redundancy. The arrays were washed, blocked and the labeled cRNA was detected by staining with streptavidin-Cy3. Hybridized arrays were scanned using an Illumina BeadStation 500X Genetic Analysis Systems scanner and the image data extracted using the Illumina GenomeStudio software, version 1.1.1.1. For statistical analysis, the expression data were filtered to include only probes with a consistent signal on each chip, and a detecton *p* value of less then or equal to 0.02 for at least one sample of the data. The resulting dataset was next analyzed with DIANE 6.0, a spreadsheet-based microarray analysis program. An overview of DIANE can be found online at http://www.grc.nia.nih.gov/branches/rrb/dna/diane_software.pdf. Using DIANE, the results were normalized with a Z-Score transformation [[3](#_ENREF_3)]. Z-normalized data were then analyzed with principal component analysis (PCA). To determine the gene expression changes within each specific RNA comparison, Z-Scores for paired treatment groups were compared using the Z-Ratio statistic [[3](#_ENREF_3)]:

$$Z-Ratio=\frac{Z-Score_{LPS}-Z-Score_{Vehicle}}{\sigma[Z-Score_{LPS}-Z-Score_{Vehicle}]}$$

Expression changes for individual genes were considered significant if they met four criteria: Z-Ratio above 1.5 or below -1.5; false detection rate (FDR)[[4](#_ENREF_4)] of less than 0.30; a *P*-value statistic for Z-Score replicability below 0.05; and mean background-corrected signal intensity greater than zero. Differentially expressed genes were identified as significant (*p* < 0.05) based on Z-scores. Gene set analysis using GO gene sets with the PAGE [[5](#_ENREF_5)] algorithm was performed as previously described [[6](#_ENREF_6)].

Microarray data has been deposited in GEO (GSE27620).

**SUPPLEMENTARY REFERENCES**

1. Chi T, Yan Z, Xue Y, Wang W (2004) Purification and functional analysis of the mammalian SWI/SNF-family of chromatin-remodeling complexes. Methods Enzymol 377: 299-316.

2. Garcia I, Mayol G, Rodriguez E, Sunol M, Gershon TR, et al. (2010) Expression of the neuron-specific protein CHD5 is an independent marker of outcome in neuroblastoma. Mol Cancer 9: 277.

3. Cheadle C, Vawter MP, Freed WJ, Becker KG (2003) Analysis of microarray data using Z score transformation. J Mol Diagn 5: 73-81.

4. Tusher VG, Tibshirani R, Chu G (2001) Significance analysis of microarrays applied to the ionizing radiation response. Proc Natl Acad Sci U S A 98: 5116-5121.

5. Kim SY, Volsky DJ (2005) PAGE: parametric analysis of gene set enrichment. BMC Bioinformatics 6: 144.

6. De S, Zhang Y, Garner JR, Wang SA, Becker KG (2010) Disease and phenotype gene set analysis of disease-based gene expression in mouse and human. Physiol Genomics 42A: 162-167.

7. Lai D, Wan M, Wu J, Preston-Hurlburt P, Kushwaha R, et al. (2009) Induction of TLR4-target genes entails calcium/calmodulin-dependent regulation of chromatin remodeling. Proc Natl Acad Sci U S A 106: 1169-1174.
